# Breath-hold imaging of the coronary arteries using quiescent-interval slice-selective (qiss) magnetic resonance angiography - pilot study at 1.5 tesla and 3 tesla

**DOI:** 10.1186/1532-429X-18-S1-P69

**Published:** 2016-01-27

**Authors:** Robert R Edelman, Shivraman Giri, Amit Pursnani, Marcos P Ferreira Botelho, Wei Li, Ioannis Koktzoglou

**Affiliations:** 1 Radiology, NorthShore University HealthSystem, Evanston, IL USA; 2 Radiology, Feinberg School of Medicine, Northwestern University, Chicago, IL USA; 3 Medicine, Pritzker School of Medicine, University of Chicago, Chicago, IL USA; 4 Radiology, Pritzker School of Medicine, University of Chicago, Chicago, IL USA; 5 Siemens Healthcare, Chicago, IL USA

## Background

Coronary magnetic resonance angiography (MRA) is usually obtained with a free-breathing navigator-gated 3D acquisition. Our aim was to develop an alternative breath-hold approach that would allow the coronary arteries to be evaluated in a much shorter time and without risk of degradation by respiratory motion artifacts. For this purpose, we implemented a breath-hold, non-contrast-enhanced, quiescent-interval slice-selective (QISS) 2D technique. Sequence performance was compared at 1.5 and 3 Tesla using both radial and Cartesian k-space trajectories.

## Methods

The left coronary circulation was imaged in six healthy subjects and one patient with coronary artery disease. Breath-hold QISS was compared with T2-prepared 2D balanced steady-state free-precession (bSSFP) and free-breathing, navigator-gated 3D bSSFP.

## Results

Approximately 10 2.1-mm thick slices were acquired in a single 20-sec breath-hold using two-shot QISS, and 20 slices using single-shot QISS. QISS contrast-to-noise ratio (CNR) was 1.5-fold higher at 3 Tesla than at 1.5 Tesla. Cartesian QISS provided the best coronary-to-myocardium CNR, whereas radial QISS provided the sharpest coronary images. QISS image quality exceeded that of free-breathing 3D coronary MRA with few artifacts at either field strength. Compared with T2-prepared 2D bSSFP, multi-slice capability was not restricted by the specific absorption rate at 3 Tesla and pericardial fluid signal was better suppressed. In addition to depicting the coronary arteries, QISS could image intra-cardiac structures, pericardium, and the aortic root in arbitrary slice orientations.

## Conclusions

Breath-hold QISS is a simple, versatile, and time-efficient method for coronary MRA that provides excellent image quality at both 1.5 and 3 Tesla. Image quality exceeded that of free-breathing, navigator-gated 3D MRA in a much shorter scan time. QISS also allowed rapid multi-slice bright-blood, diastolic phase imaging of the heart, which may have complementary value to multi-phase cine imaging. We conclude that, with further clinical validation, QISS might provide an efficient alternative to commonly used free-breathing coronary MRA techniques.

**Figure 1:**
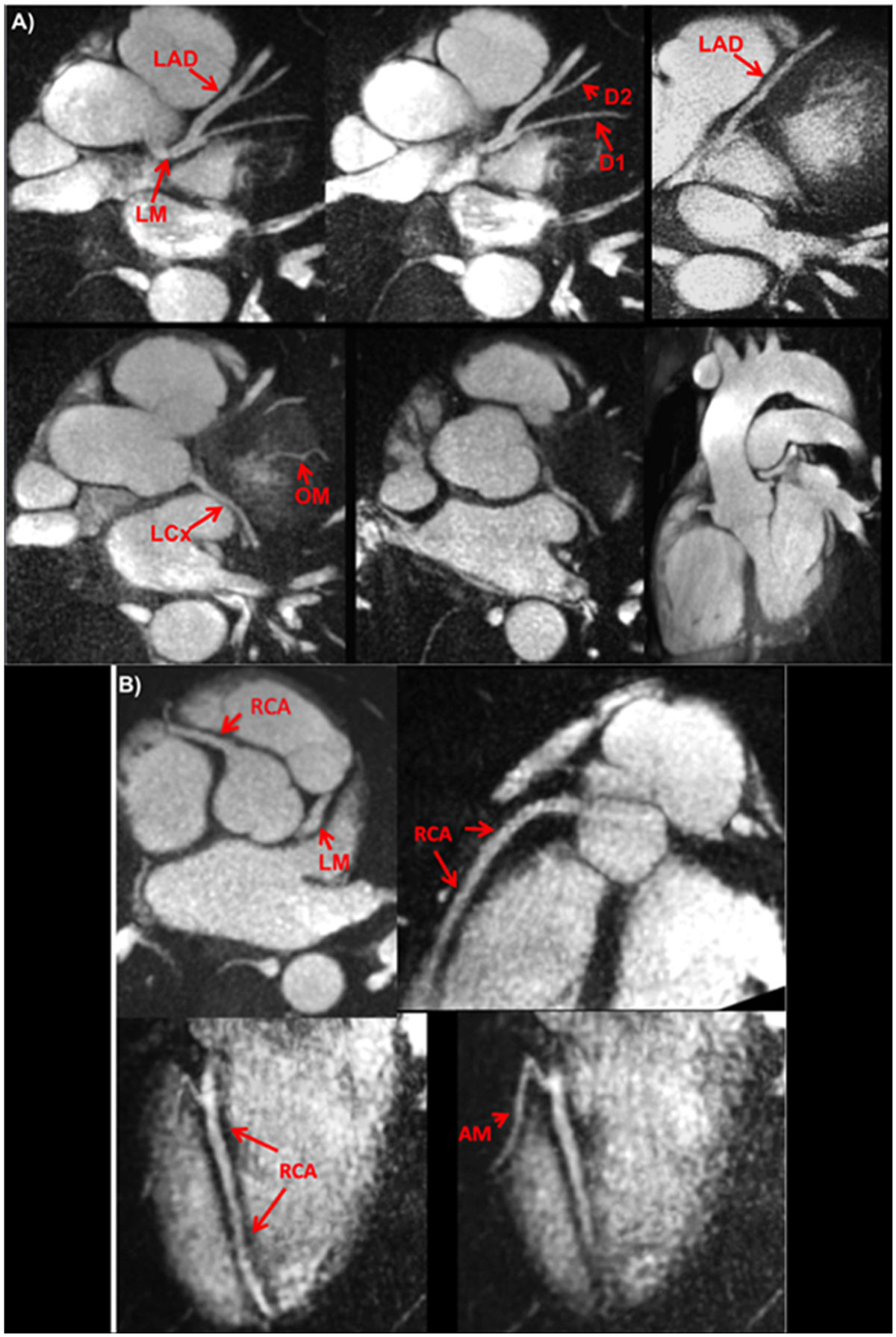
**Examples of thin MIPs reconstructed from single breath hold radial QISS**. Images were acquired at 3 Tesla using various scan orientations. A-Aorta and left coronary circulation. B-Right coronary circulation.

